# A Low Cost Matching Motion Estimation Sensor Based on the NIOS II Microprocessor

**DOI:** 10.3390/s121013126

**Published:** 2012-09-27

**Authors:** Diego González, Guillermo Botella, Uwe Meyer-Baese, Carlos García, Concepción Sanz, Manuel Prieto-Matías, Francisco Tirado

**Affiliations:** 1 Department of Computer Architecture and Automation, Complutense University of Madrid, Madrid 28040, Spain; E-Mails: dgonzalez@grupobme.es (D.G.); garsanca@dacya.ucm.es (C.G.); csanzpineda@fdi.ucm.es (C.S.); mpmatias@dacya.ucm.es (M.P.M.); ptirado@dacya.ucm.es (F.T.); 2 Department of Electrical and Computer Engineering, FAMU-FSU College of Engineering, Tallahassee, FL 32310, USA; E-Mail: umb@eng.fsu.edu

**Keywords:** computer vision, optical flow, block matching algorithm, NIOS II, very large scale integration (VLSI), FPGA, embedded systems

## Abstract

This work presents the implementation of a matching-based motion estimation sensor on a Field Programmable Gate Array (FPGA) and NIOS II microprocessor applying a C to Hardware (C2H) acceleration paradigm. The design, which involves several matching algorithms, is mapped using Very Large Scale Integration (VLSI) technology. These algorithms, as well as the hardware implementation, are presented here together with an extensive analysis of the resources needed and the throughput obtained. The developed low-cost system is practical for real-time throughput and reduced power consumption and is useful in robotic applications, such as tracking, navigation using an unmanned vehicle, or as part of a more complex system.

## 1. Introduction

The field of multimedia information has progressed very rapidly; video coding standards have become crucial when transmitting large amounts of video data. By removing temporal redundancy of video data for proper storage and transmission, motion estimation has become key for high performance in video coding. Since 1980, video coding has focused on representations of video data for storage and transmission purposes, with efficient reduction of the size of encoded video data being the most challenging issue to manage.

**Figure 1.**
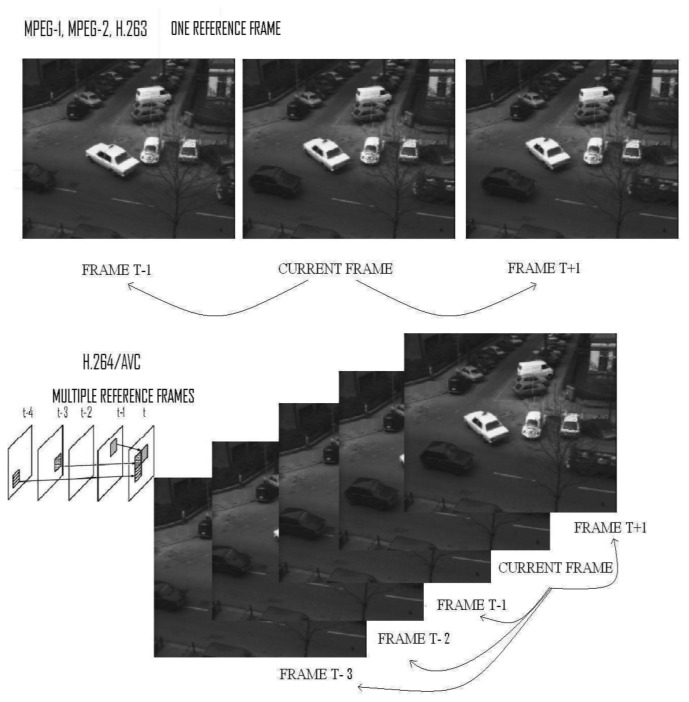
Different schemes of video compression between H.263 and H.264. H.263 (2001) encodes the motion only one reference frame at a time. Nevertheless, H.264/AVC, completed in 2004, uses multiple reference frames to encode the motion vectors as shown in the figure. It is possible to appreciate the blocks from the previous frames (t-4,t-3,t-2,t-1) projected in frame t.

The International Telecommunication Union (ITU) developed a number of video coding standards for real-time transmission applications (such as video conferencing). The first major aim of the ITU was H.261, designed for transmission over ISDN lines with data rates in multiples of 64 Kbits/s. ITU has published a series in the H.26X family, such as H.263+ [1,2]. As well, the International Organization for Standardization (ISO) and the International Electro-technical Commission (IEC) established the Moving Picture Experts Group (MPEG) in order to set standards for audio, video compression, and transmissions such as MPEG-1, MPEG-2, and MPEG-4 [3,4] (MPEG-1 aims to meet the low complexity requirement, MPEG-2 is meant for broadcast-quality television, and MPEG-4 is especially designed for low bitrate applications). In 2001, The Joint Video Team (JVT) joined the ITU-T Video Coding Experts Group (VCEG), and ISO/IEC MPEG started the development of a new video coding standard, H.264/AVC [5–7], completed in 2003. Commonly known as MPEG-4 Part 10, the standard H.264/ Advanced Video Coding (AVC) provides good video quality with lower bitrate than previous coding standards, though at the expense of notably increasing the design complexity. In earlier coding standards, such as H.261 and MPEG-1, working with one reference frame, H.264/AVC supports multiple reference frames, as Figure 1 shows.

Motion estimation plays a very important role in these video-coding standards, widely adopted in MPEG-n, H.26n. (n = 1…4). Regarding motion estimation, there are many family algorithms, strategies, and specific architecture implementations with Very Large Scale Integration (VLSI) [8,9] systems. Gradient motion estimation families are based on a constant brightness assumption.

We have developed the sensor focusing on the matching motion estimation family, which we will explain further as we consider each frame as divided into fixed-size MacroBlocks (MBs). The goal of this process is to remove temporal redundancy existing between adjacent frames by finding the Motion Vector (MV), which points up to the best macro block prediction, according to any metric in the Reference Frame (RF).

**Figure 2.**
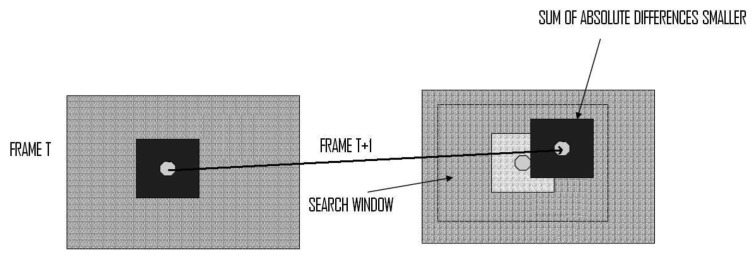
The FST scheme of the process.

This process analyzes the blocks of a reference frame, in order to estimate the closest block to the current one, as shown in Figure 2. Hence, motion vector is an offset from the coordinate of the current macro block to the corresponding macro block in the reference frame. The process of coding the frame processed with motion estimation in video is also known as inter-frame coding, which is applied to control the navigation in flying robots such as in an unmanned aerial vehicle. Motion estimation is one of the information channels to be integrated into compressed sensing in avionics.

In the framework of real-time computing sensors, there are other platforms, such as the work of Deutschmann [10] or Stocker [11,12]. Deutschmann provides an analog VLSI sensor that computes real-time division of the temporal and spatial derivatives of the local light intensity. Stocker presents a VLSI distributed visual processing sensor, with a network architecture applying an error correction strategy which is able to deliver the motion estimation components based on the Horn and Schunck gradient-model approach [13].

Regarding the matching family used in the present contribution, we reference the work of Niitsuma and Maruyama [14], who developed a high performance systolic processor system using FST. Also the University of Seoul [15] presented a matching family sensor using a NIOS II processor, although neither accuracy data (PSNR) nor throughput measures are provided. Finally Guzman *et al.* presented an embedded sensor based on a commercially specialized smart-camera [16] which is able to operate at 176 × 144 @ 10,000 fps and also uses a NIOS II processor.

The contribution of this work is a low-cost FPGA-based motion estimation sensor, which uses three selected and very well-known algorithms in the block matching family [17]. This system is designed by means of using a NIOS II soft-core microprocessor [18] and an ALTERA DE2 board [19].

The matching motion family used in this work is widely used for multimedia coding, as stated previously, the system itself is customizable, with changing the microprocessor architecture and the motion search window being possible, among other features. We have developed an analysis of the accuracy and efficiency of the system as we explain further.

This paper is organized as follows: Section 1 describes the multimedia scope and the importance of the motion estimation algorithms. Section 2 describes and specifically compares the algorithms used in the sensor functionality. Section 3 shows the hardware architecture and the primitive functions implemented and accelerated in the hardware. Section 4 discusses the results in terms of throughput and resources consumed. A visual output is shown, and a comparison with existing sensors is also accomplished. Finally, Section 5 contains the concluding remarks and future lines of this embedded system.

## 2. Matching Estimation Methods from Multimedia Video Coding Inspired by Sensor Construction

We provide, in the following paragraphs, an overview of the matching algorithms, focusing on three specific ones chosen for their peculiarities while being implemented. The aim of Block-Matching Methods (BMMs) is to estimate Motion Vectors for each Macro Block within a specific and fixed search window in the reference frame [17,20]. For example, the Full Search Technique (FST), also denoted as an exhaustive search algorithm, is one of the most straightforward methods in BMMs. The FST algorithm exhaustively matches all Macro Blocks within a search window in the reference frame to estimate the optimal Macro Block; *i.e.*, the one with the minimum Block-Matching Error (BME). There are several definitions for BME, but the most used is the Sum of Absolute Difference (SAD) of all the pixels between an MB of the current frame and that of the reference frame and the Mean Squared Error (MSE), this last metric being less conservative due to the square factor. Usually, the huge amount of computations required to calculate the error by the FST limits its applicability, turning the development of efficient motion estimation search algorithms into a significant topic for video coding.

In order to reduce the computational weight, many enhanced search algorithms have been proposed. These methods can be organized in two categories: (1) the Search Reduction (SR) of SAD and (2) the Calculation Reduction (CR) of SAD. SR algorithms are based on reducing the search points within a search window [21–24]. Examples of well-known algorithms belonging to this group are the Three-Step Search Technique (TSST) [25]; the New Three-Step Search Technique (NTSST) [26,27]; the Four-Step Search Technique (4SST) [28]; the Block-Based Gradient Descent Search Technique (BBGDST) [29]; the 2-D Logarithm Search Technique (LOGST) [30]; the cross-search algorithm [31]; the dynamic search window adjustment algorithm [32]; the Diamond Search (DS) [23]; and Hexagon-based Search (HS) algorithm [33]. These algorithms employ fixed patterns with/without limited searching steps in order to locate the MB with the minimum SAD. Many other varieties with different pattern shapes motion estimation algorithms have also been presented [34–36]. In order to accelerate the search process, we assume the target candidate points toward the inside of the local optimum; therefore the quality of the results becomes worse than with the FST. Comparison of Fast Search Techniques implemented in the presented sensor.

Conversely, algorithms categorized as CR of SAD try to reduce the computations. Since SAD is calculated by adding the differences of each pixel, the computation of the partial SAD is simpler than the computation of the total SAD between two MBs. Because of this, a Partial Distortion Search Technique (PDST) was first proposed to reduce computations in vector quantization [37]. Additionally, other techniques not addressed in this paper have been found to reduce the calculation number and improve the estimation. Several examples of this approach are the fast lossless PDS algorithm [38] or the Normalized Partial Distortion Search (NPDS) method, which rejects the invalid candidate MVs [39,40] early.

### 2.1. Full Search Technique

The Full Search Technique (FST) is the most straightforward Block Matching Method (BMM) and also the most accurate one. FST matches all possible blocks within a search window in the reference frame to find the block with the minimum Summation of Absolute Differences (SAD), defined as:

(1) $$\text{SAD}(x,y;u,v)=\frac{1}{N^{2}}\sum_{x=0}^{N-1}\sum_{y=0}^{N-1}\left|I_{t}(x,y)-I_{t-1}(x+u,y+v)\right|$$

where I_t_ (x, y) represents the pixel value at the coordinate (x, y) in the frame t and (u, v) represents the displacement of the Macro Block (MB) candidate. Thus, given a block with the size N = 32, the FS algorithm requires 1,024 subtractions and 1,023 additions to calculate a SAD. The required number of checking blocks is (1 + 2d)^2^, while the search window is limited within ±d pixels, usually by a power of two.

As seen in Figure 2, one block from the left part of Frame T is matched (using any metric error, such as the SAD) with the corresponding one from the right part of Frame T + 1 inside of the search window. The displacement from frame T to T + 1 constitutes the estimated motion for this block.

### 2.2. Three Step Search Technique (TSST) and (NSST)

The TSST [25] is the first BMM based on a non-exhaustive search. The TSST supports two important contributions for motion estimation in terms of fixed search patterns and limited search steps. Most of the later works still include these characteristics to design the algorithms.

**Figure 3.**
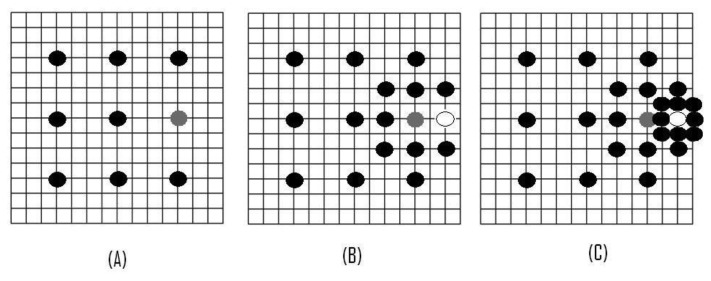
TSST. (**A**) The first step. (**B**) The second step. (**C**) The third step.

The aim here is to perform a multi-scale process, applying three steps in order to find the most similar MB within the search window of the reference frame. In the first step, the step size of the search window is designated as half of the search area. Nine candidate points, including the center point and eight checking points on the boundary of the search window, as shown in Figure 3(A), are selected in each step.

The second step moves the search center forward to the matching point with the minimum SAD of the previous step; and the step size of the second step is reduced by half, as shown in Figure 3(B). The third step stops the search process. The step size of one pixel and the optimal MV with the minimum SAD can now be obtained, as shown in Figure 3(C). Using the same search window, ±7 pixels, the TSST only needs 25 search points in comparison with the FST algorithm, which needs 255. As we can see in Table 1 FST uses more search points than TSST and 2DLog, but less search steps than these.

The new Three-Step Search Technique NTSST [27] exploits the fact that the MVs of the frame with slow motion are mostly found near the center of the search window. This technique manages a center biased checking point pattern and a halfway-stop technique for stationary MBs to improve the performance of the TSST. The process first checks the points of the pattern. If the center point contains the minimum SAD, the search is done; but if the minimum SAD appears as one of the neighbors of the center point, the NTSST checks five corners or three edge points; after this, the search is over. Otherwise, the search steps of the NTSST are similar to those of the TSST (Figure 3).

### 2.3. Two Dimensional Logarithmic Search (2DLOG) and Modifications

An alternative to the techniques previously explained is the Two Dimensional Logarithmic-based Search (2DLOG) [41], which is feasible to implement in hardware. This approach uses a pattern cross search (+) in each step, with an initial step size of d/4. The step size is reduced by half only when the minimum point of the previous step is the center one or the current minimum point reaches the search window boundary. If none of these two conditions is accomplished, the step size remains the same.

**Figure 4.**
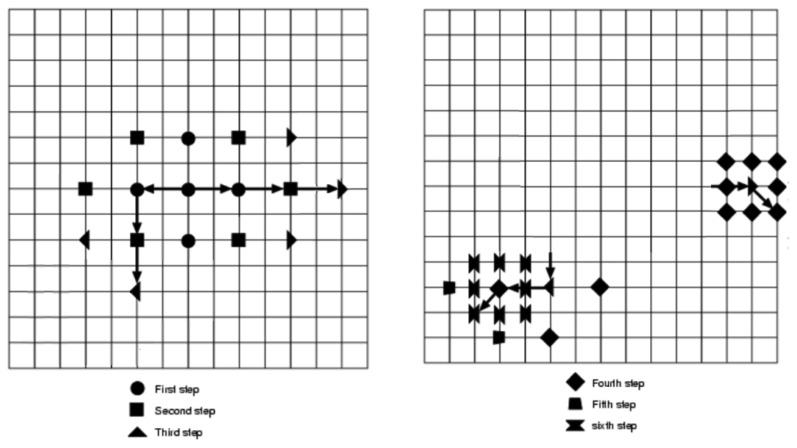
2DLOG. Two search paths for the 2DLOG search algorithm.

As an example, two different search paths are shown in Figure 4. When the step size is reduced to 1, all eight of the checking points adjacent to the center checking point of that step are searched. The bottom search pathway needs 23 = 5 + 3 + 2 + 3 + 2 + 8 checking points through the six steps to complete the process; nevertheless, the top search pathway requires 19 = 5 + 3 + 3 + 8 checking points.

## 3. Hardware Implementation of the Sensor

An embedded system [42] is a computer system which performs specific tasks and can be part of a more complex system (mechanical, optical, *etc.*). Usually this design depends on a set of parameters, such as data processing throughputs, efficiency, power consumption, reliability, configurability, and low cost, among others. Sometimes, due to the kind of application and the environment where the sensor will be used, it is desirable to keep a good trade-off solution between many of these parameters. Nowadays, many embedded systems are associated with our routine work as part of complex sensors, such as video cameras, vehicular technology, security, scientific instrumentation, optics, industrial inspection, and so on.

**Figure 5.**
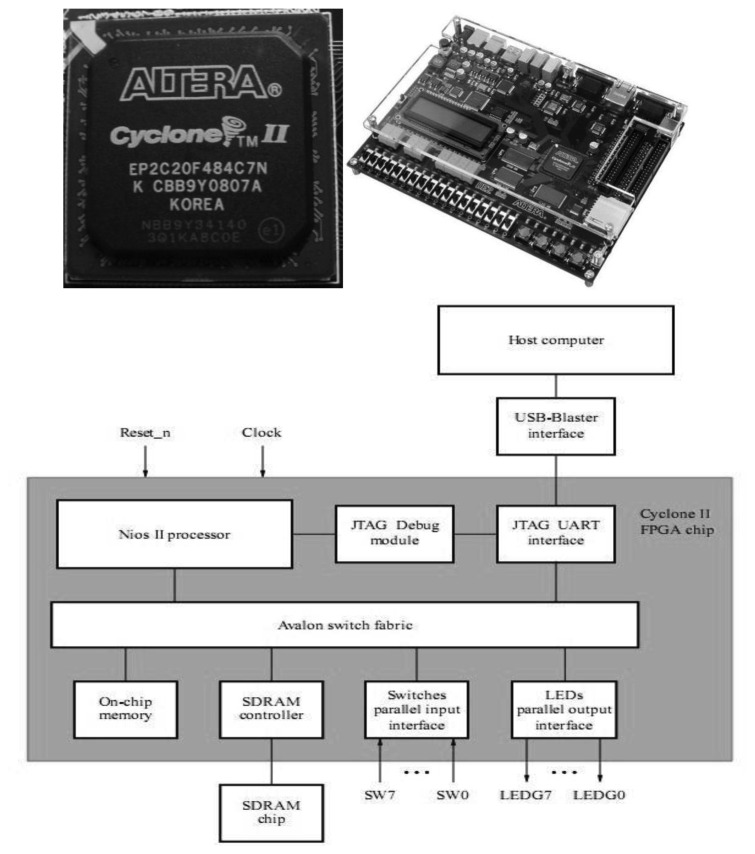
(**Top**) FPGA Chip and Prototyping Board. (**Bottom**) Cyclone II Architecture (Pictures extracted from [47]).

A Field Programmable Gate Array (FPGA) [43,44] contains millions of connections and logic cells that can be configured to achieve a specific digital logic design. FPGAs can be programmed in a large variety of low-level and high-level Hardware Description Languages (HDL) [45]. Due to the configurable capacity of the FPGA devices, a customized hardware can be designed to be included in any sensor. It is possible to design processor features, develop specialized hardware accelerators for intensive computation tasks, and create custom input/output ports to be connected with other physical parts of the sensor. These systems, built together in the same FPGA, are known today as a System-on Programmable Chip (SoPC) [46]. Figure 5 shows a real example of FPGA devices.

### 3.1. NIOS II Soft-Core Processor

The NIOS II [48] is a soft-core processor based on RISC architecture. It is targeted for Altera devices, allowing scalable development and flexibility since it can be customized with additional features depending on performance or cost objectives. NIOS II [48] is an enhanced version, which offers higher performance and a lower cost than the previous 16-bit soft-core processor NIOS [49]. This 32-bit processor belongs to a three-member family named Fast, Economy, and Standard, where each one is optimized for a specific price and performance range. Each one of the three cores uses a common 32-bit Instruction Set Architecture (ISA), with 100% binary code compatibility between them.

The NOS II/f Fast CPU is optimized for maximum performance, bringing performance up to a 220 DMIPS in the Stratix II [50] family of FPGAs, which places it squarely in the ARM 9 [51] class of processors. While this core is four times faster than the original NIOS CPU, it is 40% smaller. It has 4 K bytes of separated data and instruction cache, an oscillator of 144 MHz, and 20 embedded multipliers of 9 × 9 bits. Performance in systems based on NIOS II can scale to fit the application by means of custom instructions, high bandwidth switch fabric, and hardware accelerators. It also supports fixed and variable cycle operations. The NIOS II/e Economy CPU is optimized for the lowest cost, achieving a smaller FPGA footprint (less than 600 LEs). It has no data or instruction cache, is half the size of the smallest NIOS core, and increases performance by four times. Finally, the NIOS II/s Standard CPU is a trade-off solution between processing performance and logic element usage. It is 60% faster than the fastest NIOS CPU and smaller than the smallest NIOS CPU. It achieves over 120 DMIPS while consuming only 930 LEs (Stratix II).

### 3.2. Hardware Acceleration and Algorithms

For the current sensor, the NIOS II C2H Compiler [18] is used, moving specific functions, which are critical for performance, from running on the FPGA soft-core processor (Cyclone II EP2C35F672C6) [52] to optimized and pipelined hardware accelerators. The current accelerators have direct access to the processor's memory, largely improving the parallel transactions to the needed number of buffers.

**Figure 6.**
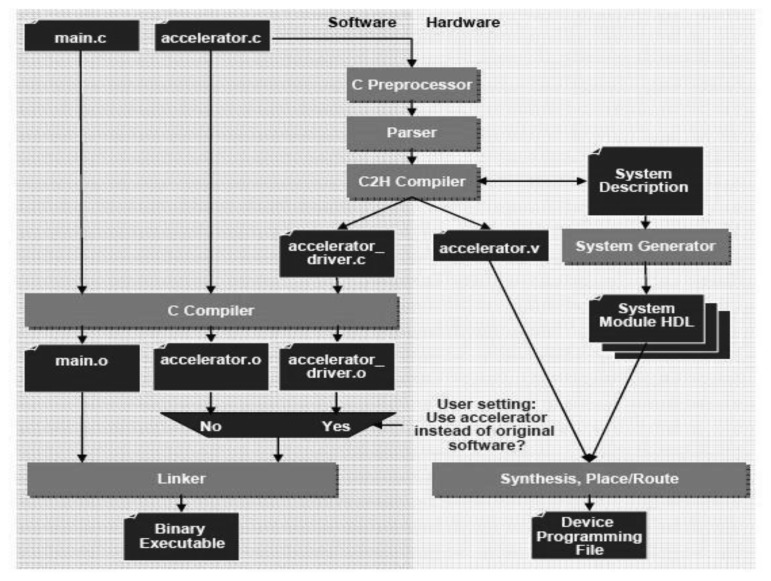

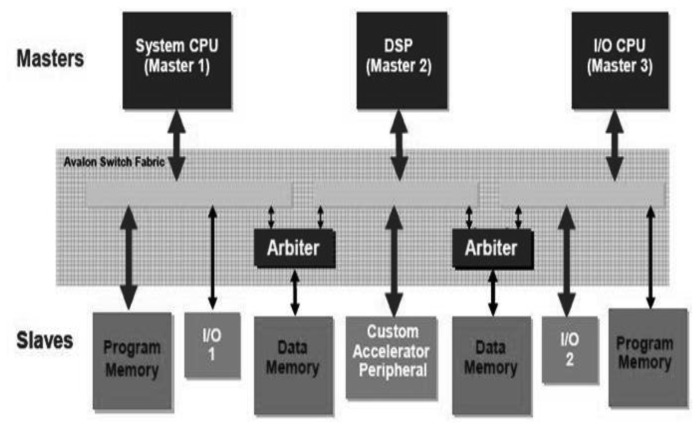
C2H Integration and Avalon Switch Fabric Connecting Master and Slave in a system (Diagram extracted from [47]).

Usually the processors share a single system bus with DMA channels and other master functions, limiting bus access to only one master. NIOS II systems benefit from the so-called Avalon Switch Fabric [53] that provides, as shown Figure 6 (right), a dedicated data path to each master, allowing all masters to transfer data simultaneously, which delivers greater system performance. This bus supports a plethora of characteristics, such as address decoding, dynamic bus sizing, clock domain crossing, off-chip interfaces, and datapath multiplexing. Large blocks of data can be processed concurrently with CPU operation constructing application-specific hardware accelerators, boosting the system throughput due to dedicated datapaths. The performance of the embedded system not only depends on the frequency or benchmarks but also on the surrounding system.

Figure 6 (up) shows how the NIOS II C2H Compiler integrates into the software build process in the IDE. The left half of the flowchart shows the standard C compilation of *main.c* and *accelerator.c*, as it occurs without acceleration. The right-hand side of the flowchart shows the hardware compilation process invoked when a function in *accelerator.c* is accelerated. It also shows the generation and selective linking of the accelerator driver into the executable file.

Altera claims that no restrictions on the bandwidth are imposed inside/outside of the accelerator different from the physical limitations of the connected memories. When the NIOS II C2H Compiler creates hardware for a function, it generates sufficient master ports for pointer and array operations. These master ports allow access to memory and other peripherals in the system and are able to operate independently, in parallel. It is also possible to write data to output buffer and fetch data from input buffers in parallel over the same clock cycle.

**Figure 7.**
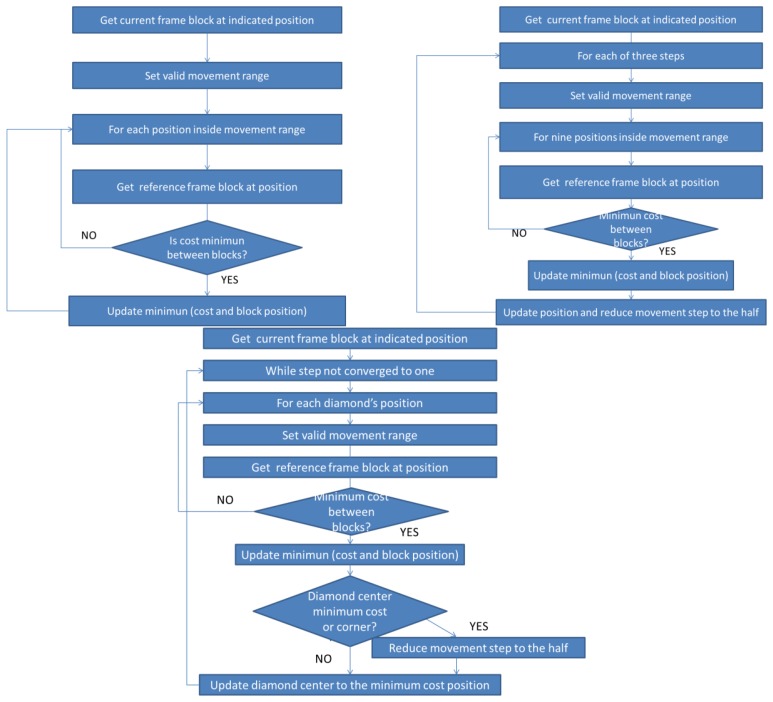
Flow Chart of Algorithms I, II, III: FST (**Upper left**): For a given block in a current frame's position, scan all blocks between a movement range on the reference frame, and compare them with the given block for achieving the minimum cost. TSST (**Upper right**): For a given block in a current frame's position, scan nine blocks (position and around) in the reference frame, and compare them with the given block for achieving the minimum cost. Then, the searching step is reduced to half, and the base position is changed to the minimum cost block one. This process is repeated three times. 2DLOG (**Lower center**): For a given block in a current frame's position, scan five blocks (diamond's center and diamond's corners) in the reference frame, comparing them with the given block for achieving the minimum cost. Then, diamond's center position is updated to the minimum cost block one or the searching step is reduced to half. This process is repeated until the searching step converges to one.

**Figure 8.**
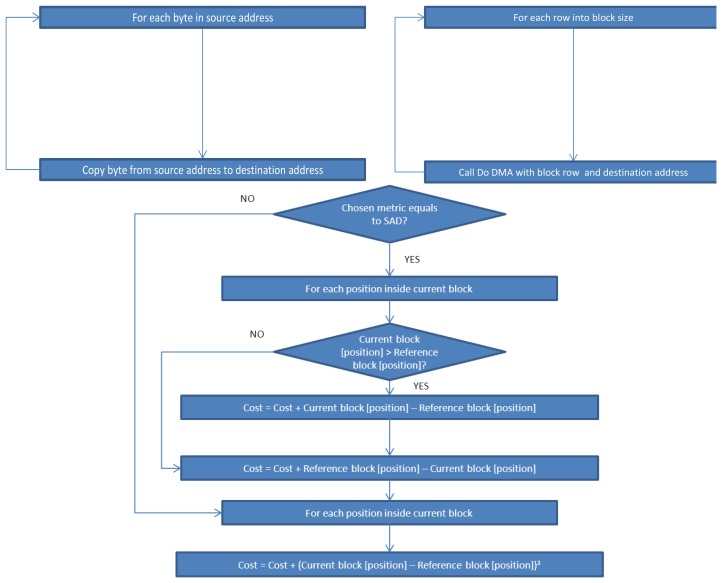
Flow Chart of Algorithms IV, V, VI: CopyDoDMA (**Upper left**): Copy “length” bytes from the source address to the destination address. GetBlock (**Upper right**): Copy one frame block into the destination address. GetCost (**Lower center**): Return cost between urrent block and reference block according to chosen metric.

Next, the functionality of the algorithms implemented in the sensor is briefly described and shown (Figures 7 and 8). The three first Algorithms (I, II, III) correspond to the techniques represented in Figures 2 through 4. Additionally, Algorithm IV moves data in memory; Algorithm V gets a specific MacroBlock (MB); and, finally, Algorithm VI delivers the accuracy of the motion estimation in the sensor itself:

- **Algorithm I.** Full Search Technique (FST). This function looks into the current frame for each block situated in (x, y) in the reference frame. All the possibilities are tested, returning the motion of the block in the current frame as explained in Section 2.1 and Figure 2.
- **Algorithm II.** Three Steps Search Technique (TSST). This function performs three steps through a limited search step and using a fixed search pattern as explained in Section 2.2 and Figure 3.
- **Algorithm III.** 2D Log Technique (2DLOG). This function performs three steps between 2 and 8 times executing a logarithmic search using a fixed search pattern as explained in Section 2.3 and Figure 4.
- **Algorithm IV.** Copy_do_DMA. This is a simple function that copies “length” bytes from the “source” memory direction to the “destiny” memory direction. It manages memory transfers.
- **Algorithm V.** Get_Block function. This function results in a copy block, pointed by “block”, of the parameter “frame”, receiving as parameters the block size, the width, and the position of the block into the frame (x, y).
- **Algorithm VI.** Get_Cost function. This function returns the cost between the current block and the reference block calculated according to a SAD metric, as shown in Equation (1).

## 4. Results Testing the Sensor

In this section, we present the results obtained applying different methods, different window searches, different processors, and also different accelerated functions.

The quality of the acceleration code is organized into four categories: (1) *no*, where the entire code is executed in the NIOS II without acceleration; (2) *low*, which accelerates do_dma (Algorithm IV); (3) *medium*, which accelerates do_dma and Get_Block (Algorithms IV & V); and (4) *high*, which accelerates all the functions (Algorithms I or II or III and IV, V, VI).

Regarding the input sequence test, we have used many well-known sequences [54] for measuring matching-based motion estimation systems, which will be commented upon later. The output sensor shows its reference motion for each Macro Block, the reference frame, and the cost expressed according to the error metric Sum of Absolute Differences or Mean Squared Error, this last metric being less conservative, as remarked upon previously.

### 4.1. Throughput Obtained

The throughput of the sensor is represented as a function of the kilopixels per second (kpps) delivered with the system. Every technique is implemented using a range of window searches of 8, 16, and 32 pixels, as well as the three different architectures of the NIOS II microprocessor (Economic, Standard, and Fast), as mentioned in Section 3.

We first considered in this preliminary analysis the behavior of the system from no code accelerated, algorithm IV, and algorithm IV+V; thus, in other words—*no*, *low*, and *medium* configurations for each one of the three matching techniques considered (FST, TSST, 2DLOG).

**Figure 9.**
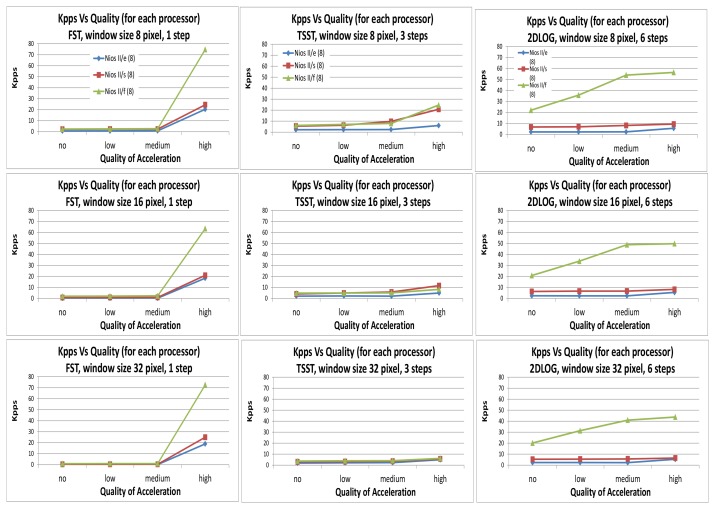
Throughput measured in kilopixels per second (kpps) obtained using FST, TSST, 2DLOG with NIOS II (e/s/f).: “**no**” runs the whole code in the NIOS II with no acceleration; “**low**” accelerates do_dma (Algorithm IV); “**medium**” accelerates do_dma and Get_Block (Algorithms IV and V); “**high**” accelerates all functions (Algorithms I or II or III and IV, V, VI together).

The throughput of the whole system with *low* or *medium* acceleration behaves similarly when comparing the execution of the whole functions in embedded software (*no* acceleration) under the NIOS II and the FST technique, as shown in Figure 9. If we focus on the TSST technique, this behavior becomes lineal (when considering *no*, *low*, and *medium* acceleration). If we see the 2DLOG technique, the linear response is emphasized again for *no*, *low*, and *medium* acceleration, as well as the fast architecture (NIOS II /f). Although every throughput for every architecture depends on the window size (8, 16, and 32 pixels, respectively), the linear tendency of all responses remains constant for all sizes. We notice, for instance, that by using only *medium* acceleration (Algorithms IV and V), the 2DLOG technique and fast architecture (NIOS II /f) is obtained as throughput range between 16 and 21 Frames per second (Fps) at a 50 × 50 pixel resolution when using different windows sizes.

Focusing on *high* acceleration (Algorithms I or II or III and IV, V, VI together), we can appreciate a different throughput regarding the windows size: Regarding FST (in the first column of Figure 9) and fast architecture, a range from 61 to 72 kpps is delivered, depending on the window size used (from 8 to 32 pixels). This is a throughput for the system between 24.5 and 29 fps at a 50 × 50 pixel resolution, enough for a small sensor camera. For configurations of standard and economic architecture, we obtain a throughput range between 20 and 27 kpps (from 8 to 32 pixels), which is a range between 8 and 11 fps at 50 × 50 pixel resolution.

Focusing on TSST (in the second column of Figure 9) and regarding fast architecture, a range from 6.15 to 24.6 kpps is delivered, depending of the window size used (from 8 to 32 pixels). This means a throughput for the system between 2 and 10 fps at a 50 × 50 pixel resolution. For configurations of standard and economic architecture, we obtain a throughput range between 5 and 20 kpps (from 8 to 32 pixels) which means a range between 2 and 8 fps at a 50 × 50 pixel resolution.

The 2DLOG technique (in the third column of Figure 9) processes a range from 43.8 to 56.4 kpps for fast architecture, again depending on the window size used (from 8 to 32 pixels), which means a range of 17.5–22.5 fps. For configurations of standard and economic architecture, we obtain a throughput of approximately 10 kpps and 8 kpps, independent of the window range (from 8 to 32 pixels), which means a range between 2 and 8 fps at a 50 × 50 pixel resolution.

Note that the size of the window is not always inversely proportional to the system throughput. For example, the TSST restricts the calculation complexity by limiting the exhaustive search to three steps, so accelerating all functions means a trade-off solution between pixel parallel level (the increment of the window size involves less Macro Blocks) and Macro Block parallel level (when window size is decreased).

### 4.2. Used Resources

The hardware resources used are listed in Table 2 (Full acceleration) and Table 3 (*no* acceleration, *low* acceleration, and *medium* acceleration) for different processor architectures, a window size of 32 pixels, and a set of different architectures.

Recall the cache resources regarding the microprocessor: no cache (economic), only data cache (standard), and both data and instruction cache (fast). The tables show the number of Logic Cells (LCs) used, the number of embedded multipliers (9 × 9) needed, and the total number of bits.

High quality acceleration (all functions) requires a little bit less than 50% of the available logic cells (35%–39% for FST, 40%–44% for TSST and 39%–44% for 2DLOG). In this case, between 33% and 39% of embedded DSPs (9 × 9) are used and total memory Bits (Block Rams) are from 9% to 24%, depending on the motion estimation technique considered.

Regarding *low*, *medium*, and *no* accelerations, note that the same resources are required for the three techniques, although it depends on the processor configuration selected. Focusing on *medium* quality, we obtain an increment from 15% to 20% of Logic Cells for NIOS II *economic* to NIOS II *fast*. Regarding the multipliers, this increment is from 17% to 23% for NIOS II *economic* to NIOS II *fast*. Finally, regarding the total memory bits, the increment covers from 9% to 24% for NIOS II *economic* to NIOS II *fast*.

Focusing on *low* quality, the resources used are 10%–15% (Logic Cells), 0%–6% (Multipliers), and 9%–24% (Total Memory bits). Regarding the *no-acceleration*, we obtain an increment 6%–11% (Logic Cells), 0%–6% (Multipliers), and 9%–24% (Total Memory bits). These two increments are the same as the *low* quality; in other words, constant DSPs and Block Ram from the previous configuration is maintained.

### 4.3. Resources vs. Performance

**Figure 10.**
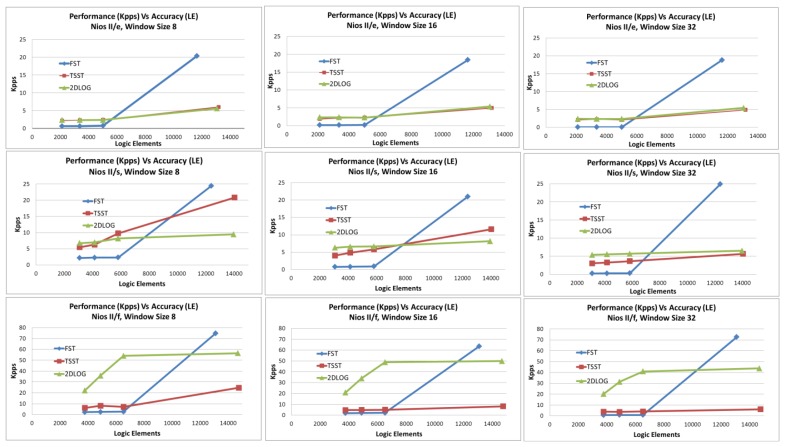
Performance in kilopixels per second (kpps) versus logic elements applied obtained using FST, TSST, 2DLOG with NIOS II (e/s/f). The four measures correspond to the four types of acceleration (*no*, *low*, *medium* and *high*).

**Figure 11.**
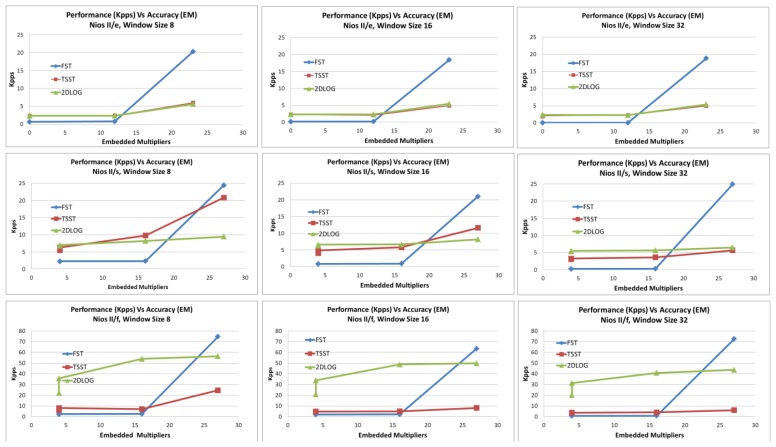
Performance in kilopixels per second (kpps) versus embedded multipliers using FST, TSST, and 2DLOG with NIOS II (*e/s/f*). The four measures correspond to the four types of acceleration (*no*, *low*, *medium*, and *high*).

In order to compare used resources and performance, we show the kilopixels per second (kpps) achieved versus the logic elements implied and the embedded multipliers implied for each design in Figures 10 and 11.

We can appreciate how every window search has been distinguished and every processor type discussed on the previous point. For every graph and for every processor kind, we have measured four points, which belong to every kind of acceleration tested on this approach.

As we can observe, for NIOS II/e and NIOS II/s processors, FST algorithm achieves less kpps with the same logic elements than with TSST and 2DLOG in all acceleration types, except in the case of high acceleration in which FST achieves better performance than any other using less logic elements.

Regarding the sensor design, when the NIOS II/f processor is used, we can see 2DLOG gets the best performance and the TSST achieves better performance than the FST in all acceleration types, except on high acceleration. In this last case, FST obtains the best results using less logic elements, followed by 2DLOG and, finally, by TSST.

Using the NIOS II/e processor, *no* acceleration and *low* acceleration on a desired design achieves the same results, spending no embedded multipliers. When chosen acceleration is *high*, all algorithms use the same quantity of embedded multipliers, although FST achieves the best performance.

When NIOS II/s is selected, the FST gets the worst results whether or not the design is *no* accelerated or accelerated in *low* mode; but when accelerating in *high* mode, the FST gets the best results using the same resources. Using this processor, the TSST gets better results than the 2DLOG in all cases except on high acceleration with a window size of 32. As the window size increases, the more the TSST decreases its difference against 2DLOG.

As we can observe, the FST and 2DLOG are the best designs using NIOS II/f, but 2DLOG gets better results using *no* acceleration or *low* acceleration, and FST achieves better results using the *high* acceleration mode. The TSST only can be compared with the FST in either *no* acceleration or *low* acceleration modes, where they gets the same results, due to 2DLOG achieving better results than TSST in all cases.

### 4.4. Block Matching Accuracy (PSNR)

In order to measure the accuracy of the sensor, we use the Mean Squared Error (MSE), similar to Equation (1) but using the absolute value of the squared subtractions, becoming less conservative metrically, which emphasize the larger differences:

(2) $$\text{MSE}(x,y;u,v)=\frac{1}{N^{2}}\sum_{x=0}^{N-1}\sum_{y=0}^{N-1}{\left|I_{t}(x,y)-I_{t-1}(x+u,y+v)\right|}^{2}$$

Next, we can define the Peak-Signal-to-Noise-Ratio (PSNR) as shown in Equation (3), where *Max_value* refers to the peak-to-peak value of the original data, which depends on the frame-grabber or the camera datasheet used. In this case, the *Max_value* corresponds with an 8-bit range, so an intensity value of 256. This value characterizes the motion compensated image created by using motion vectors and macro clocks from the reference frame:

(3) $$\text{PSNR}(x,y,u,v)=20\log_{10}\left[\frac{\text{Max}\_\text{value}}{\sqrt{\text{MSE}}}\right]$$

**Figure 12.**
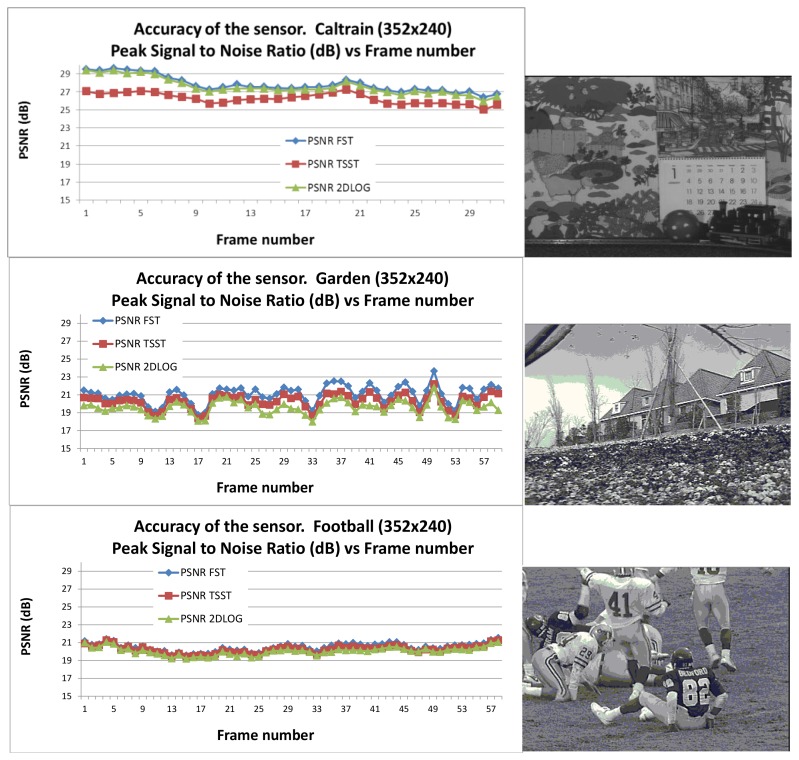
Accuracy of the sensor under different algorithms (FST, TSST, 2DLOG) using the “Caltrain,” “Garden,” and “Football” sequence [54].

The value of the PSNR for three different sequences as *Caltrain*, *Garden*, and *Football* deeply used for testing motion estimation sequences [54] with a resolution of 352 × 240 pixels (4:2:0 and SIF format) as shown in Figure 12. As is evident, the accuracy of the implemented FST configuration remains the highest for the three cases. The TSST and 2DLOG configurations alternate in terms of accuracy, the difference between the three implementations lower than 2 dB.

### 4.4. Visual Results and Other Sensor Approaches

**Figure 13.**
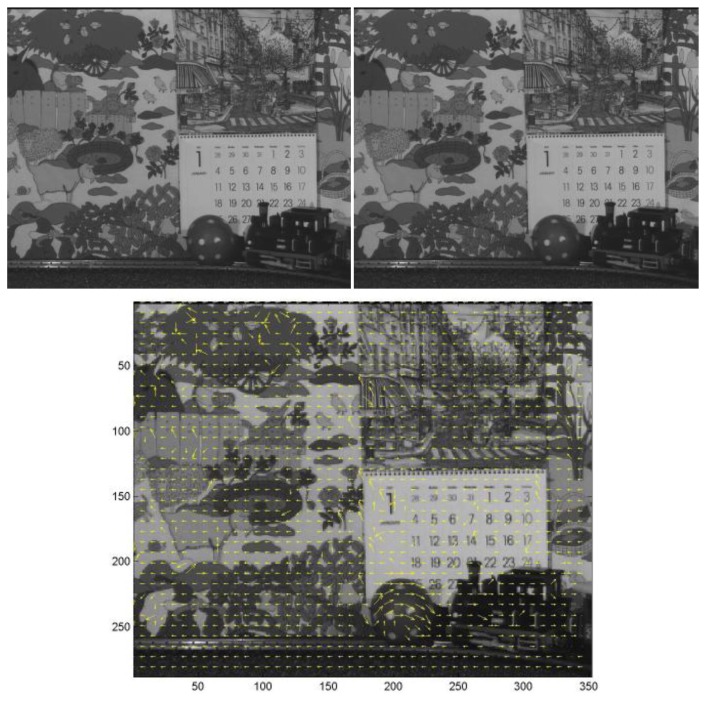
(**Top**) Two consecutive frames of the test stimuli [54]. (**Bottom**) Motion Estimation calculated with the sensor implementation when using FST.

Next, we briefly show some visual results delivered by the platform. We can see an example of two frames from the Caltrain sequence [54] corresponding to 352 × 288 pixels (CIF format), as shown in Figure 13. The yellow arrows show the motion estimation superposed within the reference frame. If we just apply the motion compensation, it is trivial to subtract the motion vectors from the current frame and transmit only the motion difference, emulating the MPEG/H.26x compression process flow as indicated in Section 1.

In the framework of real-time computing sensors, there are other platforms (commented upon in the introduction), where family, chips used, and performance results have been represented in Table 4.

Regarding the two most frequently and hardware-implemented family methods for recovering motion estimation: On one hand, we have the differential or gradient methods derived from work using the image intensity in space and time. The speed is obtained as a ratio from the above measures [8,9,16]. On other hand, we have correlation-based methods, frequently used in this contribution. The methods work by comparing positions from the image structure between adjacent frames and inferring the speed of the change in each location, probably the most intuitive methods [28–30].

Regarding the chip used, the many approaches include: (1) Full Custom VLSI, as a methodology for designing integrated circuits by specifying the layout of each individual transistor and the interconnections between them [42]; (2) Altera Cyclone and Cyclone II as 130-nm and 90-nm FPGAs to provide a customer-defined feature set for high-volume, cost-sensitive applications [52]; and (3) XC2V6000—a 150-nm FPGA [57] and NIOS II [18], further commented upon in Section 3.

### 4.5. Performance Conclusions

Comparing used resources and obtained performance, we can extract some conclusions from our approach and guide the designer through different ways for achieving his preferred goal. The designer will be able to choose one option depending on his priorities--powerful designs, low cost designs, or efficient designs.

- Powerful design: NIOS II/f processor running an FST algorithm accelerating at a high level using window sizes 8 or 32, which achieves the best performance.
- Efficient design: NIOS II/f processor running a 2DLOG algorithm accelerating at a medium level using a size 8 window, which achieves good performance without significant hardware cost.
- Low Cost design: NIOS II/e processor running an FST, TSST, or 2DLOG without accelerating the design and using any window size, because any of these use the least possible resources.

## 5. Conclusions

The present approach describes a low-cost sensor in an embedded platform, using the Altera C2H in order to accelerate Block Matching Motion Estimation Techniques. Regarding motion compensation, this technique is useful for multimedia, image stabilization in robotic and unmanned vehicles, and recently, for 4-D medical imaging. The NIOS II processor allows the creation of a plethora of devices, such as SDRAM, UART, SRAM and a custom instructions device, all while embedding everything in a processor by means of an Altera SOPC builder. This methodology approach reduces the peripheral hardware design's complexity, enhancing the development of System on Chip.

This sensor has been also characterized in terms of accuracy with the usual PSNR metric for matching systems, resulting in a stable framework that suggests using the FST mode when maximum accuracy is required. At the same time, it is the most hardware resource consuming configuration, wasting about 40% for LEs and embedded DSPs and 25% of Block Ram memory. This system is able to deliver 72.5 kpps, equivalent to a SOC, which processes 50 × 50 @ 29.5 fps.

Future research lines include plans to integrate a full binocular disparity (stereo matching) method together with the presented motion estimation sensor in an embedded system in order to calculate 3D motion. We plan to extend this system with a larger FPGA than the one used here and test the whole system in a little robot, autonomous vehicle, or similar structure. In this way, we would have an affordable solution for accelerating matching algorithms while keeping a trade- off between accuracy and efficiency.

## Figures and Tables

**Table 1.** Comparison of Fast Search Techniques implemented in the presented sensor.

**Method**	**Number of Search Points**		**Number of Search Steps**	
	
	**MIN**	**MAX**	**MIN**	**MAX**
FULL SEARCH (EXHAUSTIVE)	225	225	1	1
THREE-STEP SEARCH	25	25	3	3
2D-LOG SEARCH	13	26	2	8

**Table 2.** FPGA resources measured with a Quartus tool [19] with a window size of 32 pixels. Case “h” (high quality acceleration). Processors “e” and “s” and “f” mean NIOS II/ “economic”, “standard”, and “fast”.

**Processor/Quality**	**Method**	**Logic Cells**	**Method**	**Logic Cells**	**Method**	**Logic Cells**	**(FST,TSST,2DLOG)**	

							**EMs (9 × 9)**	**Total memory bits**
			
e/h	FST	11,637 (35%)	TSST	13,173 (40%)	2DLOG	13,056 (39%)	23 (33%)	44,032 (9%)
s/h		12,382 (37%)		14,023 (42%)		13,955 (42%)	27 (39%)	79,488 (16%)
f/h		13,090 (39%)		14,755 (44%)		14,678 (44%)	27 (39%)	114,944 (24%)

**Table 3.** FPGA resources measured with a Quartus tool [19] with a window size of 32 pixels for either FS, TSST, or 2DLOG. Processor “e” and “s” and “f” means NIOS II/ “economic”, “standard”, and “fast”. Case “n” and “l” and “m” mean *no*, *low*, and *medium* quality acceleration, respectively.

	**Processor e**			**Processor s**			**Processor f**		
			
**Quality**	**Logic Cells**	**EMs (9 × 9)**	**Total memory bits**	**Logic Cells**	**EMs (9 × 9)**	**Total memory bits**	**Logic Cells**	**EMs (9 × 9)**	**Total memory bits**
n	2107 (6%)	0 (0%)	44032 (9%)	3085 (9%)	4 (6%)	79488 (16%)	3763 (11%)	4 (6%)	114944 (24%)
l	3363 (10%)	0 (0%)	44032 (9%)	4147 (12%)	4 (6%)	79488 (16%)	4889 (15%)	4 (6%)	114944 (24%)
m	5006 (15%)	12 (17%)	44032 (9%)	5812 (17%)	16 (23%)	79488 (16%)	6524 (20%)	16 (23%)	114944 (24%)

**Table 4.** Summary of Throughput (kilopixels/s) for prior sensors. NP means “Not Provided”.

**Models**	**Family**	**Chip used**	**Throughput (kilopixels/s)**	**Image Size (pixel)**	**Image Rate (frame/s)**

Present work	Matching	Altera Cyclone II EP2C35F672C6/ NIOS II	72.5	50 × 50	29
Deutchmann 1 *et al.* [10] (1998)	Gradient H&S [13]	Full Custom VLSI	0.12	20	5
Stocker 2 *et al.* [11,12] (2006)	Gradient H&S [13]	Full Custom VLSI	5.1	30 × 30	6
Niitsuma *et al.* [14] (2006)	Matching	Xilinx XC2V6000	9200	640 × 480	30
Yong Lee *et al.* [15] (2008)	Matching	Altera Cyclone EP1C20F400C7/ NIOS II	NP	NP	NP
Guzman *et al.* [55] (2010)	Gradient L&K [56]	NIOS II / Eye-RIS [16]	729	176 × 144	28.8

1 Considering a pixel size 147 μm × 270 μm and maximum rotational velocity of 353 rpm.

2 Taking into account a maximum Bias = 0.67 Volts.
